# Cardiac Platypnea-Orthodeoxia Syndrome: A Rare Case of Flow-Directed, Right-to-Left Shunt via a Patent Foramen Ovale Exacerbated by Aortic Root Enlargement

**DOI:** 10.7759/cureus.43721

**Published:** 2023-08-18

**Authors:** Victor H Molina-Lopez, Porfirio E Diaz-Rodriguez, Eric Aviles-Rivera, Juan Vazquez-Fuster, Josue Mercado-Crespo, Sonia Vicenty-Rivera, Claudia Rosales

**Affiliations:** 1 Cardiology, Veterans Affairs Medical Center, San Juan, PRI

**Keywords:** intracardiac shunt, pfo occluder device, patent foramen ovale closure, patent foramen oval, cardiac plathypnea-othodeoxia

## Abstract

Cardiac platypnea-orthodeoxia is a unique clinical syndrome characterized by dyspnea and deoxygenation when moving from a supine to an upright position. In this case report, we detail the experience of a 78-year-old male with persistent hypoxemia following a paradoxical embolic ischemic stroke. Despite proper management of his respiratory symptoms, the patient continued to be affected by marked dyspnea and hypoxemia, particularly when upright or in a right-sided decubitus position. Subsequent investigation revealed that his hypoxemia was a result of cardiac platypnea-orthodeoxia syndrome (POS). This condition was attributed to the enlargement of his aortic root and ascending aorta, coupled with a counterclockwise rotation of the heart axis. These factors facilitated a flow-directed, right-to-left interatrial shunt through a patent foramen ovale, even in the absence of elevated right heart pressures.

## Introduction

Cardiac platypnea-orthodeoxia syndrome (POS) is an interesting clinical syndrome characterized by dyspnea and deoxygenation upon changing from a supine to an upright position. For POS to occur, it requires two components: a primary anatomical component for interatrial communication and a secondary functional component that causes positional deformation of the atrial septum, redirecting interatrial flow when assuming an upright position [1-3]. The primary anatomical component can be an atrial septal defect (ASD), a patent foramen ovale (PFO), or an aneurysm of the atrial septum (ASA) with fenestrations. Secondary functional components lead to positional alterations in the blood flow within the heart chambers. These changes can arise from conditions like pericardial effusion or constrictive pericarditis; pulmonary disorders, such as those following a pneumonectomy; or vascular causes, such as an aortic aneurysm or elongation of the ascending aorta [1]. This case report presents a patient with cardiac POS attributed to the aortic root and ascending aorta enlargement, leading to a right-to-left intracardiac shunt via a PFO in the absence of elevated right heart pressures. Additionally, we delve into the cardiovascular etiologies linked with POS and explore the diagnostic and therapeutic strategies available for its management.

## Case presentation

We present the case of a 78-year-old male evaluated for an unexplained cause of hypoxemia. He had a history of hypertension, diffuse B-cell lymphoma on palliative chemotherapy, and remote history of recurrent past transient ischemic cerebral events. He had a prior evaluation with a transthoracic echocardiogram (TTE) with an intracardiac shunt identified on an agitated saline bubble test, which led to the diagnosis of a PFO by transesophageal echocardiography (TEE) (Video 1). He was on guideline-directed secondary stroke prevention and low-dose aspirin monotherapy. About eight months later, the patient was readmitted with hypoactivity, dysarthria, and right-sided weakness. Head computerized tomography (CT) images revealed an acute embolic infarction at the left internal capsule. The patient was provided intravenous (IV) thrombolytics acutely. In addition, he was found with peripheral oxygen saturation (SaO2) of 70% while receiving 100% FiO2 supplemental oxygen via a non-rebreather mask while in a semi-sitting position. Due to decreased mentation and hypoxemia, he was mechanically ventilated.

**Video 1:** TEE on modified mid-esophageal short axis view demonstrating the right-to-left shunt of agitated saline infused through an upper extremity peripheral vein. Note the differences in flow from the IVC and SVC and the effect of the prominent Eustachian valve on the redirection of flow. TEE, transesophageal echocardiogram; IVC, inferior vena cava; SVC, superior vena cava

A physical exam initially revealed hypoactivity, right-sided weakness, and dysarthria, which improved after IV thrombolytics with minor residual deficits and complete recovery of his alertness. On heart auscultation, there were no murmurs, S3 or S4. Lungs were clear to auscultation. There was no perioral cyanosis, acrocyanosis, or peripheral edema. The 12-lead electrocardiogram (ECG) revealed normal sinus rhythm and was essentially normal. No atrial fibrillation or flutter was identified on telemetry monitoring. Bilateral deep vein thrombosis (DVT) was identified on ultrasound, which was attributed to a progressively worsening sedentary lifestyle and B-cell lymphoma. The patient had been complaining of progressive dyspnea on minimal exertion and when assuming an upright position, for which he had been restricting his activities and spending most of the day bedbound. Given the history of a PFO, the ischemic stroke was considered secondary to a paradoxical embolism from a provoked lower extremity DVT. He was started on apixaban with low-dose aspirin for antithrombotic therapy.

**Figure 1:**
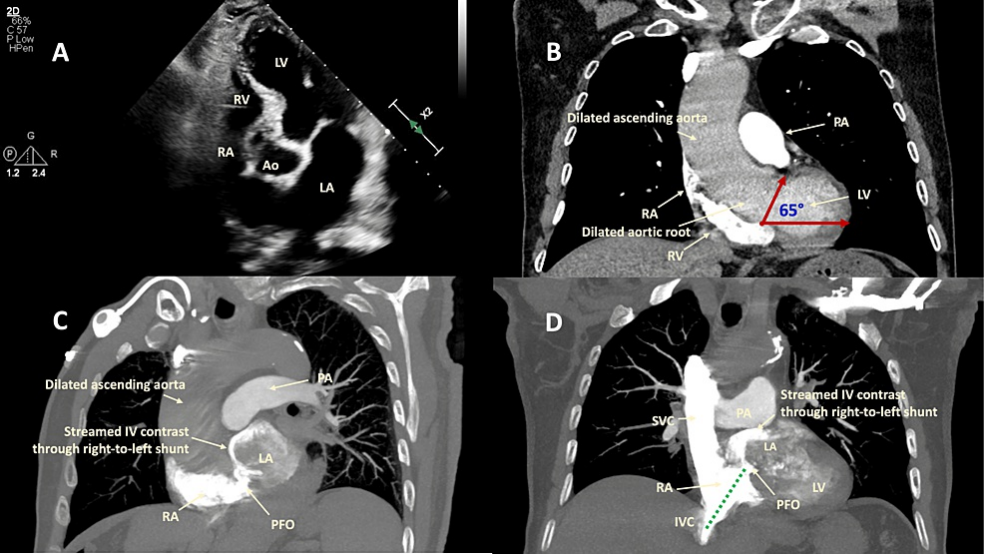
TTE and contrast-enhanced chest CTA with pulmonary embolism protocol. (A) TTE apical five-chamber view with dilated aortic root and ascending aorta covering the RA. The RV appears of normal size without any structural signs of pulmonary hypertension. The heart has an offset axis with counterclockwise rotation secondary to the dilated aortic root and the ascending aorta. Chest CTA image with pulmonary embolism protocol in coronal (B and D) and oblique (C) views showing the horizontal alignment of the aortic root and fusiform ascending aortic aneurysm (increased aortic angulation of 65°), reorienting the PFO with the inflow from the IVC (green dotted line). Streaming of intravenous contrast from the RA into the LA is visualized due to the flow-directed, right-to-left interatrial shunt despite normal right heart chambers (C and D). TTE, transthoracic echocardiogram; CTA, computed tomographic angiography; PFO, patent foramen ovale; RA, right atrium; LA, left atrium; Ao, aortic root; RV, right ventricle; LV, left ventricle; PA, pulmonary artery; SVC, superior vena cava; IV, intravenous

However, the hypoxemia persisted, requiring a high-flow nasal cannula (HFNC) after withdrawal from mechanical ventilation. A limited TTE was performed to evaluate for cardiac function, which revealed normal-sized left and right ventricles, preserved left ventricular ejection fraction, and no signs of pulmonary hypertension. However, there was aortic root dilation, and the heart had an offset axis notable on the apical views, limiting the visualization of the right ventricle and atrium (Figure 1A). Right-to-left interatrial shunting of agitated saline on the first three cardiac cycles was consistent with the known PFO. 

The patient underwent evaluation for pulmonary embolism with a chest CT angiography (CTA), which was negative for embolism with normal pulmonary parenchyma. No arteriovenous malformations were noted. Moreover, the aortic root was dilated 4.7 cm × 4.7 cm with fusiform ascending aorta dilation of 4.8 cm × 4.7 cm (Figures 1B-1C) and increased aortic angulation of 65° (Figure 1B). IV contrast flowed from the right to the left atrium through the PFO (Figures 1C-1D).

Considering no other obvious explanations for the patient’s hypoxemia, he was evaluated for plathypnea-orthodeoxia. Hypoxemia was noted to be worse while sitting or lying on the right side. Arterial blood gas (ABG) analysis revealed an arterial SaO2 of 98% while supine or in the left lateral decubitus position, with HFNC delivering 100% FiO2 at a flow rate of 40 L O_2 _per minute. However, when the bed was inclined to 50°, ABG SaO2 dropped to 87% with the onset of dyspnea. Fortunately, it gradually returned to 98% after the patient resumed the supine position for three minutes. Dyspnea was severe enough to prevent the patient from assuming a standing position. No peripheral signs of cyanosis were evident on the physical exam.

Because of the normal right heart chambers on TTE, ventilation/perfusion scintigraphy (V/Q scan) was performed to evaluate for an extracardiac shunt; however, none was identified. The cardiac right-to-left shunt was of Qp:Qs of 1.06. Right heart catheterization with oximetry run was performed to evaluate for pulmonary hypertension and shunt fraction, consistent with the VQ scan, both performed in a supine position. The right-to-left shunt was 1.3% while supine on a non-rebreather mask. Left atrial pressures were normal (11 mmHg) on invasive measurement through the PFO and higher than the right atrial pressures (8 mmHg).

**Figure 2:**
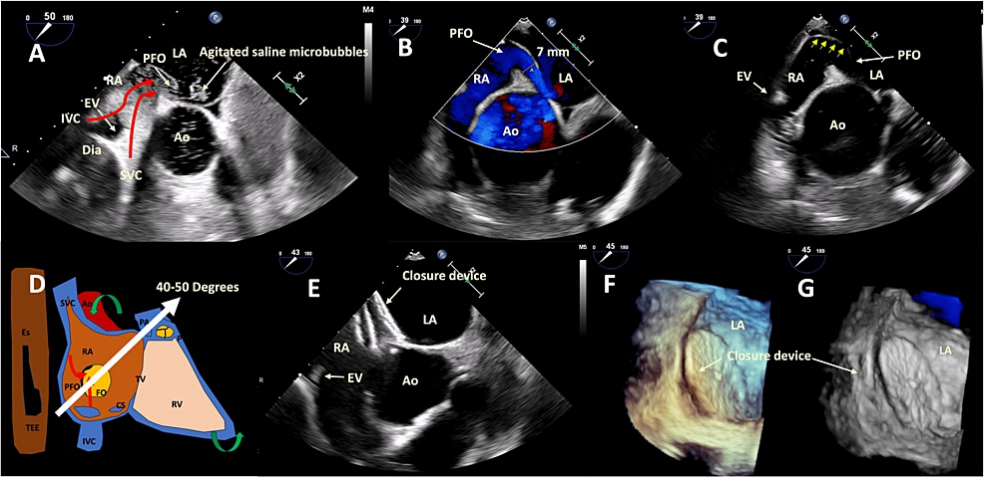
TEE images in modified mid-esophageal views depicting the flow-directed, right-to-left shunt through the PFO. (A) Initial TEE with agitated saline via an upper extremity peripheral vein, revealing a large PFO with a right-to-left shunt. This TEE was done about eight months before the images were obtained in (B) through (G). Note the marked increase in the ascending aorta size from (A) 3.5 cm × 3.5 cm to (B) and (C) 4.8 cm × 4.7 cm. The flow-directed, right-to-left shunt can be appreciated through the PFO on color flow Doppler. (C) Maneuvers that increase venous return through the right side of the heart can lead to a *spinnaker* effect toward the aneurysmal septum (yellow arrows), keeping it open and worsening the intracardiac shunt. (E) Occlusive device (Abbott Amplatzer 30 mm PFO Occluder, Abbott Park, IL, USA) after placement, (F) seen on three-dimensional TEE reconstruction with (G) color flow Doppler demonstrating the absence of a residual shunt. TEE, transesophageal echocardiogram; PFO, patent foramen ovale; RA, right atrium; LA, left atrium; Ao, aorta; SVC, superior vena cava; IVC, inferior vena cava; EV, Eustachian valve; PA, pulmonary artery; TV, tricuspid valve; Es, esophagus; CS, coronary sinus

A pre-procedural TEE re-demonstrated the enlarged and horizontally positioned aorta and aortic root with a prominent Eustachian valve. Loosening of the atrial septum was noted, allowing the oval fossa valve to move freely like an atrial septal aneurysm in normal right and left atrial pressures. Therefore, the oval fossa valve had a *spinnaker* effect with the increased venous flow (e.g., Valsalva maneuver), bulging toward the left atrium, keeping the oval foramen widely open. The Eustachian valve enhanced the effect and redirected the inferior vena cava (IVC) flow toward the PFO (Figures 2A-2C; Video 2). 

**Video 2:** TEE images in a modified mid-esophageal short-axis view demonstrate the effects of the enlarged aortic root on the PFO and the flow-directed, right-to-left shunt. An enlarged aorta can compress atrial structures, loosening the tension in the atrial septum and allowing the oval fossa valve to move more freely under normal atrial pressures, resembling an atrial septal aneurysm. Consequently, the oval fossa valve experiences a *spinnaker* effect with the venous flow, causing it to bulge toward the left atrium and keeping the oval foramen open. TEE, transesophageal echocardiogram; PFO, patent foramen ovale

**Figure 3:**
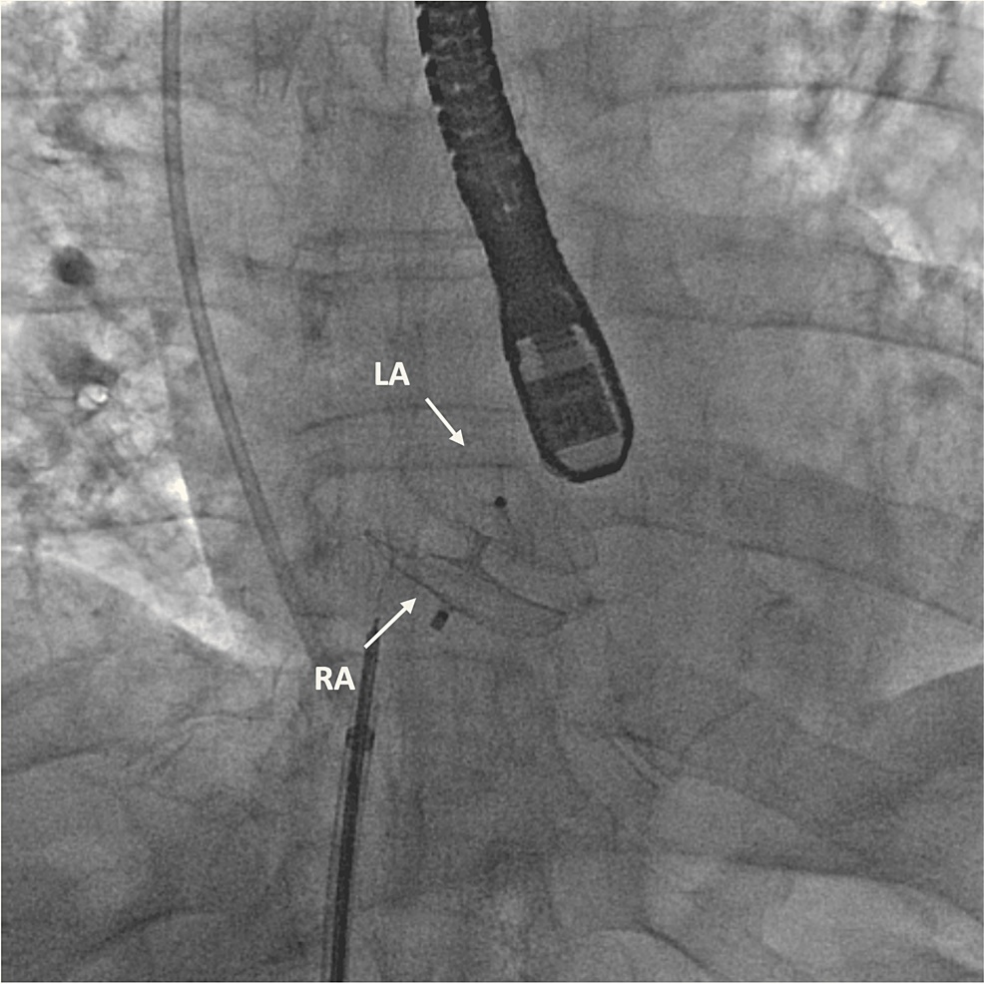
TEE- and fluoroscopy-guided PFO occluder device implantation Fluoroscopic image of the Abbott Amplatzer 30-mm PFO occluder device in interatrial septum with an anteroposterior view with arrows pointing to the left and right atrial disc deployed in the interatrial septum. TEE, transesophageal echocardiogram; PFO, patent foramen ovale; RA, right atrium; LA, left atrium

In this case, the patient met the criteria for PFO closure due to paradoxical embolic stroke and POS and was referred for fluoroscopic and TEE-guided percutaneous closure of the PFO (Figure 3). An Abbott Amplatzer (Abbott Park, IL, USA) 30-mm PFO occluder was implanted (Figures 2C-2D) without residual right-to-left shunt by color flow Doppler (Figure 2E). Following the procedure, a postprocedure TTE was conducted, indicating no signs of residual shunt. After a few days, the patient experienced relief from symptoms and discontinued supplemental oxygen. The patient was recommended to continue apixaban and low-dose aspirin for three to six months and then low-dose aspirin for life. During the one-month follow-up, TTE revealed a well-positioned closure device with no residual shunt.

## Discussion

In patients with POS, 87% present with intracardiac communication between the two atria as the primary cause. Extracardiac causes are less common, accounting for intrapulmonary arteriovenous malformations (9.2%) and lung parenchymal diseases (3.7%) [1]. POS typically stems from right-to-left shunting at the interatrial level, necessitating both an anatomical and functional anomaly that alters the anatomical axis while maintaining normal right heart pressures [1-2]. This defect often manifests as an ASD, a PFO, or less frequently, an ASA with fenestration. Although most patients with an isolated PFO do not display a right-to-left interatrial shunt due to the left atrium's higher pressure facilitating functional closure, the PFO itself is an anatomical passage letting blood flow between the infoldings of the right atrial wall (septum secundum) and the flap valve of the fossa ovalis (septum primum) [1,4,5].

POS in the absence of elevated right heart pressures can occur due to specific functional cardiac or pulmonary abnormalities that preferentially direct blood flow through interatrial communication without a pressure gradient [1,3,6,7,8,9]. In flow-directed, right-to-left interatrial shunts, the pressure in the right heart remains unaltered. Typically, in healthy individuals, blood from the superior vena cava (SVC) flows downward in the anterior portion of the right atrium, whereas blood from the IVC flows upward in the posterior portion of the right atrium. However, a change in cardiac anatomy can cause a redirection of blood flow from the IVC, favoring entry into the left atrium through interatrial communication [1].

Cardiac POS can present in patients with various conditions, such as aortic aneurysm, aortic root dilatation, and aortic elongation [10-14]. Additionally, it has been observed in individuals with a persistent Eustachian valve or a Chiari network [14,15]. It is worth noting that tricuspid regurgitant flow can also drive blood directly into the left atrium through interatrial communication [16-18]. Some patients with congenital interatrial communication might develop symptoms of POS only in late adulthood, especially those with a degenerative and age-dependent process such as aortic root dilatation and ascending aortic aneurysms. In our patient’s case, he had been free of symptoms until late adulthood. His functional right-to-left shunt worsened as the aortic root and ascending aorta enlarged, changing the heart axis and leading to progressive dyspnea and hypoxemia.

When aortic abnormalities are present, intracardiac shunting through the PFO can occur due to multiple mechanisms. An enlarging aortic root and ascending aorta lead to right atrial compression and counterclockwise heart rotation, distorting the normal atrial septum morphology. This displacement shifts the oval fossa rightward and narrows the angle between the atrial septum and the IVC. Moreover, the aortic root may protrude above the tricuspid valve orifice, exhibiting characteristics like an acquired *cor triatriatum dexter*, especially when accompanied by a Eustachian valve. Furthermore, the enlarged aortic root results in the relaxation of interatrial septum tension, enabling the oval fossa valve to move more freely under normal atrial pressures, resembling an atrial septal aneurysm. As a result, the oval fossa valve experiences a *sailboat* or *spinnaker* effect with the venous flow from the IVC and SVC, causing a bulging effect toward the left atrium, which maintains the oval foramen widely open (Video 2) [1]. Additionally, the aortic arch may positionally compress the SVC [1,3,10]. These anatomical irregularities are particularly prominent in the right lateral decubitus position and while sitting, which helps sustain interatrial communication [19-20].

Detecting POS can be challenging as the diagnosis often presents with inconspicuous signs. However, obtaining clues to the disorder can be achieved through a thorough patient history and maintaining a high level of clinical suspicion. Patients may exhibit positional variations in oxygen saturation with postural changes. Oxygen saturation should be analyzed in both supine and upright positions. An oxygen saturation that decreases by more than 5% in the upright position, with an improvement when assuming the supine position, should raise the possibility of POS [1]. Heightened suspicion should arise if hypoxemia fails to improve with 100% FiO2, thus suggesting the presence of a clinically significant right-to-left shunt. Dyspnea experienced in the upright position that disappears when assuming a lying position, along with orthodeoxia (sPO2 < 90% or pO2 < 60 mmHg in the upright position, normalization in the lying position), may also serve as additional indicators [1].

The subsequent phase involves pinpointing the root cause of desaturation. Given that cardiac etiologies are the most prevalent factors contributing to POS, the initial step in the diagnostic process should entail an echocardiogram with contrast utilizing IV agitated saline. Furthermore, this examination should be conducted in both supine and upright positions. The study's findings can aid in distinguishing patients with intracardiac shunting from those with extracardiac shunting based on microbubbles of agitated saline in the left atrium within the first three cardiac cycles, suggesting an intracardiac shunt [1,21]. Delayed opacification of microbubbles in the left atrium (after three to six cardiac cycles) suggests extracardiac shunting, usually in the pulmonary vasculature [7]. The agitated saline test through the femoral vein should be considered, when possible, to assess the presence of preferential flow from the IVC into the interatrial communication. The agitated saline solution in the upper extremity enters the right atrium via the SVC; however, patients with a prominent Eustachian valve in the IVC may have blood flow directed toward the interatrial septum and will be demonstrated in the bubble study through the femoral vein [1].

If a TTE yields inconclusive results, a TEE can be conducted to visualize the interatrial communication directly. TEE is also valuable for pre-procedural planning when contemplating shunt closure. In cases where a high suspicion remains despite an indeterminate echocardiographic study, cardiac magnetic resonance imaging (MRI) can assess anatomical distortions that may lead to right-to-left shunting [22]. To evaluate for intrapulmonary shunting, a V/Q scan can help assess for extrapulmonary uptake [23]. Perfusion scintigraphy, which employs technetium-99m-labeled macroaggregated albumin, can help assess changes in lung perfusion during positional changes (upright vs. supine) and estimate the shunt fraction [24]. If an echocardiogram suggests extracardiac shunting, a chest CTA can be another tool to help evaluate for pulmonary arteriovenous malformations [25].

The primary treatment for POS resulting from an intracardiac shunt is addressing the inherent cardiac anomaly. This often involves procedures like PFO closure or surgical repair of interatrial communication. While surgery has been a longstanding method for closing interatrial communications, percutaneous closure offers reduced mortality at a lower cost for suitable anatomies [1,2]. This technique employs a septal occlusion device, and patients typically exhibit significant symptom improvement post-procedure, boasting favorable long-term safety and efficacy [1,26-28]. Notably, our case met the criteria for PFO closure due to both PFO-associated stroke and POS. For patients aged 60 or older with a prior PFO-associated stroke, the Society for Cardiovascular Angiography and Interventions (SCAI) guideline panel recommends PFO closure over prolonged antiplatelet therapy alone [29].

## Conclusions

In conclusion, POS should be suspected in patients with unexplained dyspnea and hypoxemia, given the subtle nature of the symptoms and their positional relationship. A thorough and meticulous evaluation is necessary to uncover this uncommon and elusive diagnosis to guide the appropriate interventions. The treatment approach should be multidisciplinary. Available treatment alternatives involve medical, percutaneous, and surgical approaches. They have the potential to alleviate symptoms and even achieve a cure. With a comprehensive approach, significant improvement in the quality of life of patients affected by POS can be achieved.
